# Intelligent diagnosis of thyroid nodules with AI ultrasound assistance and cytology classification

**DOI:** 10.3389/fendo.2025.1546983

**Published:** 2025-05-21

**Authors:** Xiaojuan Cai, Ya Zhou, Jie Ren, Jinrong Wei, Shiyu Lu, Hanbing Gu, Weizhe Xu, Xun Zhu

**Affiliations:** Department of Breast and Thyroid Surgery, The Second Affiliated Hospital of Soochow University, Suzhou, China

**Keywords:** thyroid nodule, artificial intelligence, ultrasonography, cytology, indeterminate thyroid nodules

## Abstract

**Objective:**

Accurate evaluation of thyroid nodules is crucial for effective management; however, methods such as ultrasonography and Fine Needle Aspiration Cytology (FNAC) can be subjective and operator-dependent. Indeterminate thyroid nodules (ITNs) complicate diagnosis, coming at the expense of time, money, and potentially additional FNA samplings, causing more discomfort for the patients. Recent advancements in artificial intelligence (AI) assisted ultrasound diagnosis system have demonstrated excellent diagnostic performance and the potential to aid in the differentiation of ITNs. This study aims to develop an AI classifier that integrates the AI-assisted ultrasound diagnosis system, FNAC, and demographic data to enhance the differentiation of benign and malignant thyroid nodules, and to compare the diagnostic performance of the models, with a focus on diagnosing ITNs.

**Materials and methods:**

In the present research, 620 thyroid nodules were collected from a single medical center and divided into training and testing cohorts (Testing1). We developed five AI models using distinct classification algorithms (Logistic Regression, Support Vector Machine, K-Nearest Neighbor, Random Forest, and Gradient Boosting Machine) that integrate demographic data, cytological findings, and an AI-assisted ultrasound diagnostic system for thyroid nodule assessment. These models underwent prospective validation (Testing2, n = 243) to identify the optimal model. A subsequent prospective study (Testing3) involving 70 thyroid nodules further evaluated the model’s performance, where the selected optimal model was compared against FNAC combined with BRAF V600E mutation analysis.

**Results:**

After validation with the Testing1 and Testing2 cohorts, the Random Forest (RF) model demonstrated the best overall performance among the five classifiers. The area under the curve (AUC) for the RF model to diagnose thyroid nodules was 0.994 in the training cohort, 0.993 in the testing cohort, and 0.977 in the prospective data. In addition, for 42 included ITNs in the prospective data, the accuracy, sensitivity, and specificity of the RF model were 90.48%, 89.47%, and 91.30%, respectively. In the Testing 3 cohort, the RF model demonstrated superior diagnostic performance compared to both the standalone AI ultrasound auxiliary diagnostic system and FNAC alone. Its accuracy was comparable to FNAC combined with BRAF V600E mutation analysis. Conclusion: Our developed thyroid nodule AI diagnostic model shows favorable predictive value. It can serve as a decision support tool for non-thyroid specialists and assist thyroid surgeons in the management of ITN.

**Conclusion:**

Our developed thyroid nodule AI diagnostic model shows favorable predictive value. It can serve as a decision support tool for non-thyroid specialists and assist thyroid surgeons in the management of ITN.

## 1 Introduction

Thyroid cancer incidence has surged significantly in recent decades, with an estimated 821,173 new cases globally in 2022, ranking seventh among malignant tumors (1). Considering that surgery is the mainstay treatment, accurate preoperative assessment of thyroid nodules by a specialist thyroid surgeon is crucial for managing patients with suspicious thyroid lesions. Thyroid surgeons in resource-limited regions, alongside non-thyroid specialists, should have access to a dependable and cost-efficient approach for discriminating between benign and malignant thyroid nodules. This would aid in guiding referrals or surveillance.

In clinical practice, ultrasonography plays a pivotal role in thyroid screening (2). Suspicious nodules identified through ultrasonography undergo Fine Needle Aspiration Cytology (FNAC) (3), with results classified using The Bethesda System for Reporting Thyroid Cytopathology (TBS) (4). This system stratifies nodules into six diagnostic categories. Despite FNAC’s moderate diagnostic accuracy (sensitivity 86%, specificity 71% (5)), diagnostic uncertainty persists for indeterminate thyroid nodules (ITNs), particularly TBS-3 and TBS-4 nodules that show a risk of malignancy (ROM) from 13 to 34%. Notably, while the 2023 TBS update classifies TBS-5 nodules (ROM from 67 to 83%) as ITNs, this study focuses on TBS-3 and TBS-4 nodules where clinical management dilemmas are most pronounced. ITNs require further molecular testing or diagnostic surgery. Although molecular testing is less invasive than surgery, it faces limitations in cost-effectiveness and diagnostic variability. In the U.S., Afirma GSC (sensitivity 91%, specificity 68%) and ThyroSeq v3 (sensitivity 94%, specificity 82%) are cost-prohibitive for routine use (6). China predominantly employs BRAF V600E mutation analysis, which demonstrates low sensitivity (21%) and restricted applicability to TBS-3 or TBS-5 nodules (7, 8). Both molecular testing and surgical resection impose significant financial burdens, with molecular diagnostics remaining inaccessible in resource-limited regions (9).

The application of artificial intelligence (AI) in thyroid nodule has expanded exponentially (10). Machine learning algorithms, such as logistic regression and support vector machines, have demonstrated remarkable performance in the analysis of well-defined medical data. Deep learning based on convolutional neural networks, has enabled automated feature extraction and pattern recognition from medical images, uncovering diagnostic information that may be imperceptible to human analysis (11). The AI ultrasound auxiliary diagnosis system, based on deep learning, has been the most extensively studied and widely researched in the field of thyroid nodule diagnosis, employing standardized mathematical algorithms to minimize inter-observer variability (12–14). Its diagnostic performance is comparable to, or even superior to, that of sonographers (15–17). A meta-analysis has suggested that ultrasound is helpful in differentiating TBS-3 nodules (18). Additionally, some researchers have investigated the diagnostic accuracy of the AI-assisted ultrasound diagnosis system for ITNs, finding it comparable to that of BRAF V600E mutation analysis (19). This provides a reference for using the system to assist in the diagnosis of ITNs.

The overall purpose of the present study is to provide a more cost-effective, time-saving, and accurate approach to thyroid nodule evaluation. We developed a suitable machine learning model for thyroid nodule classification by integrating the AI-assisted ultrasound diagnosis system, cytology, and demographic data. Furthermore, we aim to assess the diagnostic performance of the models we developed, particularly their efficacy in diagnosing ITNs, and to compare their performance with that of FNAC combined with BRAF V600E mutation analysis.

## 2 Materials and methods

### 2.1 Patients

**Figure 1:**
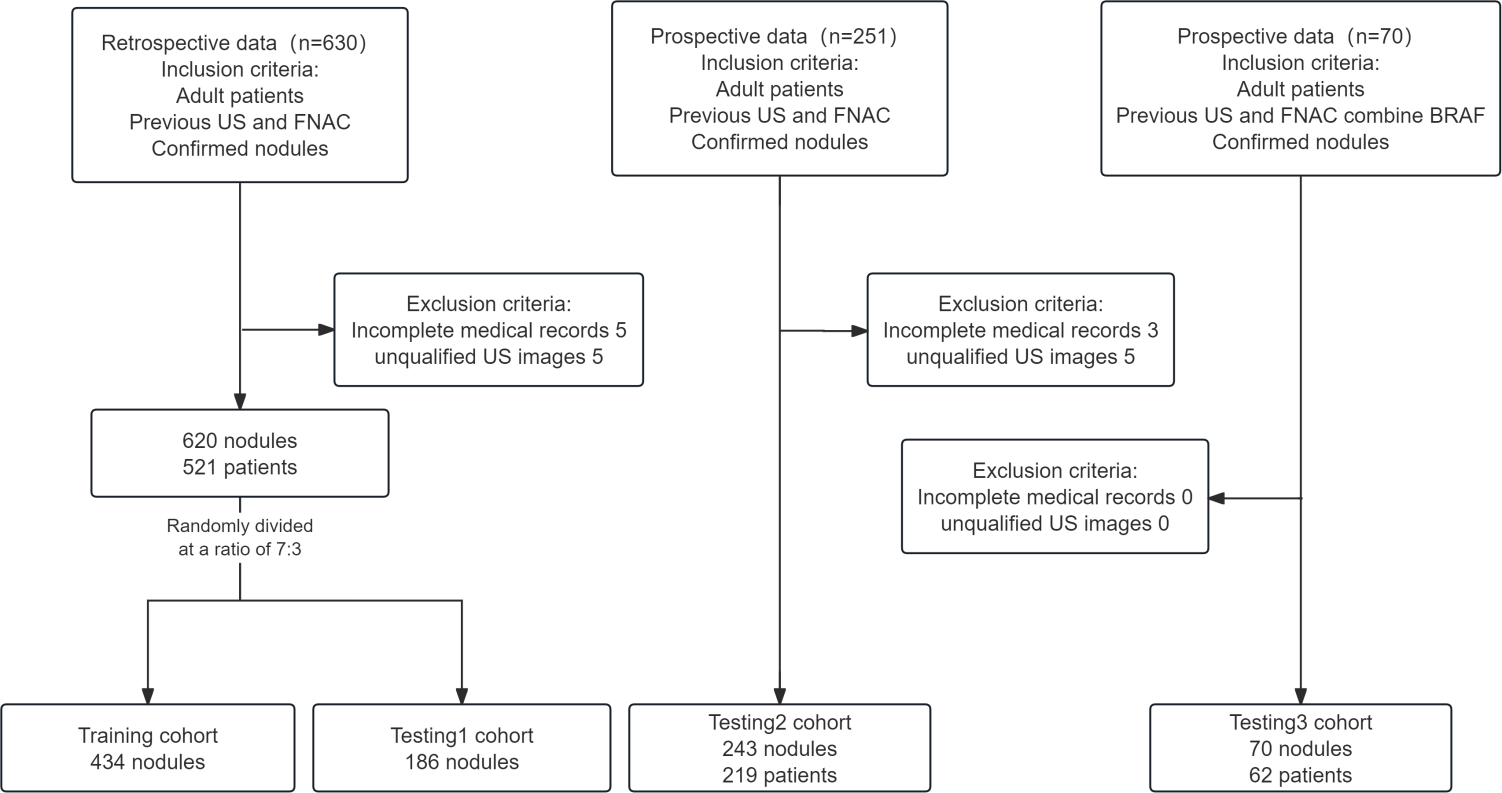
Flow chart showing patient enrollment.

Our study retrospectively collected and analyzed the ultrasound images and clinical data of patients with thyroid nodules who were admitted to the Second Affiliated Hospital of Soochow University between June 2022 and March 2024, as well as prospective data from April 2024 to September 2024. Additionally, we incorporated another prospective dataset from April 2024 to December 2024, in which each thyroid nodule had BRAF V600E mutation analysis result. This clinical study included only those patients with nodules who met all the following criteria: (1) aged 18–75 years, regardless of gender; (2) previous FNAC for thyroid nodules; (3) nodules confirmed through post-surgical histopathology or by two consistent FNAC results taken six months apart. Patients were excluded from this study if they met any of the following criteria: (1) incomplete medical records; or (2) poor-quality images (including missing ultrasound images or images where the nodules were too large to fit in a single frame). **Figure 1** illustrates the patient enrollment flowchart.

### 2.2 AI-assisted ultrasound diagnosis system

**Figure 2:**
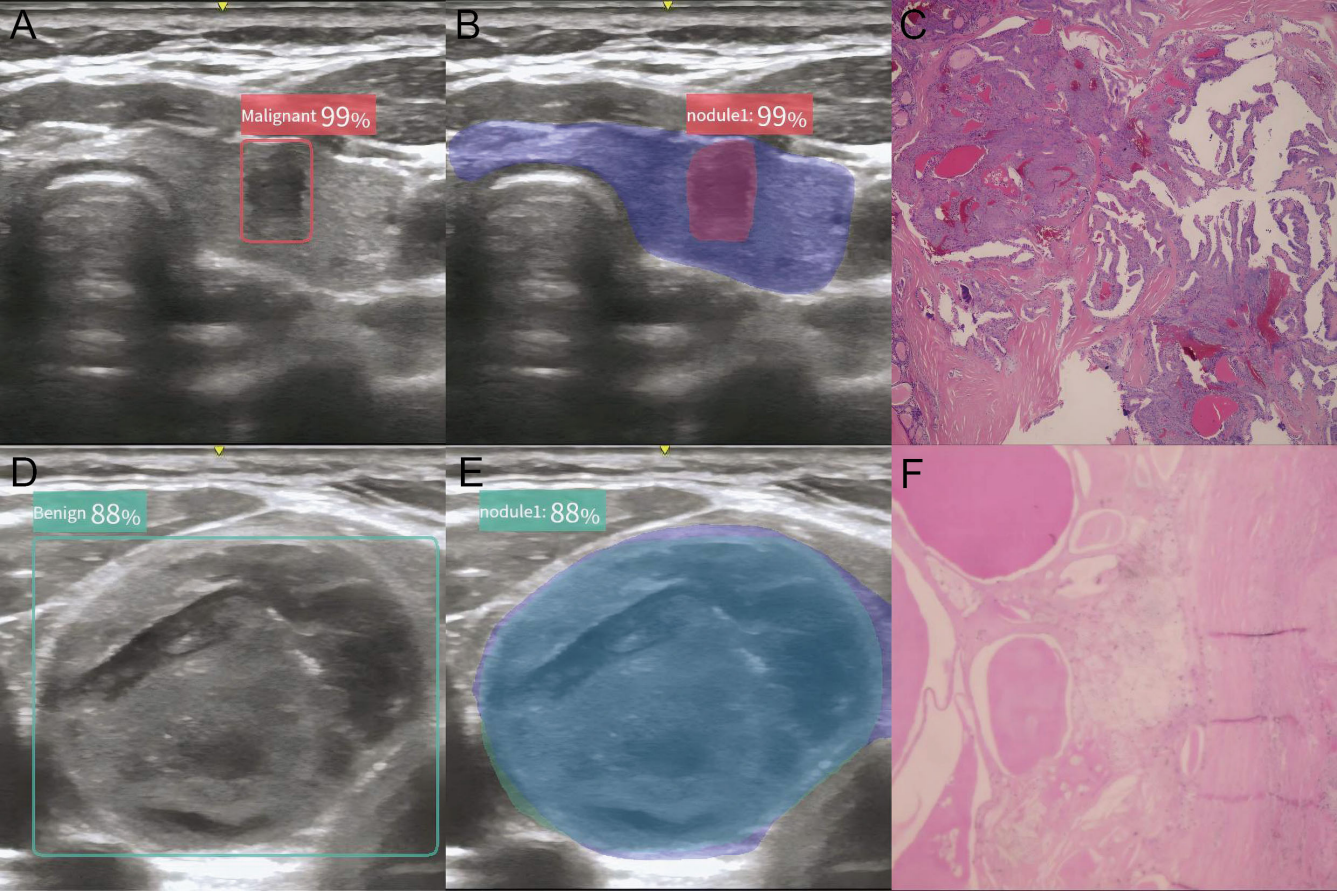
Typical plot of the AI-assisted ultrasound diagnosis system. **(A, B)** AI diagnostic plot of malignant thyroid nodule; **(C)** Histology of malignant thyroid nodule; **(D, E)** AI diagnostic plot of benign thyroid nodule; **(F)** Histology of benign thyroid nodule.

Ultrasound imaging devices equipped with high-frequency (4–12 MHz) linear-array probes were routinely utilized in this analysis. All ultrasound images of the nodules were captured and archived according to the protocol specified in the ACR Thyroid Imaging, Reporting and Data System (TIRADS) (3). The AI-assisted ultrasound diagnostic system employed in this study was the Ian Thyroid Solution 100 (MedAI Technology Co. Ltd., Wuxi, China). The ITS100 is capable of automatically detecting lesions and predicting the malignancy rate. It employs computer vision and convolutional neural network technologies to create an AI-assisted diagnostic model for identifying benign versus malignant thyroid nodules. After extracting and analyzing features from the input ultrasound image of thyroid nodules, the ITS100 will output two types of predicted value: one for malignancy and one for benignity. If the predicted value of malignancy is no less than the predicted value of benignity, the nodule is diagnosed as malignant, as demonstrated by a red ‘Malignant’ sign. Conversely, if the predicted value of benignity is higher, the nodule is diagnosed as benign, marked by a green ‘Benign’ sign (**Figures 2A–F**). For simplification, when the green ‘Benign’ sign appears, the malignant probability is calculated as 100% minus the benign probability. No calculation is required for the red ‘Malignant’ sign.

### 2.3 FNAC diagnosis of thyroid nodules

The FNAC of thyroid nodules followed The Expert Consensus and Operational Guidelines for Ultrasound-Guided Fine Needle Aspiration Biopsy of Thyroid Nodules (2018 Edition) (20). For patients with nodules suspected to be malignant based on routine ultrasound and neck physical examination, FNAC was conducted subsequently. The patient was in a supine position with their neck fully exposed. Under ultrasound guidance, the appropriate puncture site was selected, and a 25G needle was used to perform a puncture at the center of the targeted nodule. The needle tip was introduced and gently moved in a to-and-fro motion 2–3 times within different regions of the nodule. Following this, the needle was swiftly withdrawn and the collected tissue was processed for cytological examination. All procedures were performed by a single operator. Although this research utilized TBS (2017 Edition) in the FNAC reports (21), the 2023 edition of TBS (4) introduced a ‘single name’ for each of the six diagnostic categories to reduce confusion. Therefore, we applied the classification names from the 2023 Edition to our research: Class I, Nondiagnostic; Class II, Benign; Class III, Atypia of Undetermined Significance; Class IV, Follicular Neoplasm; Class V, Suspicious for Malignancy; and Class VI, Malignant. Based on the ROM and management recommendations in TBS, we assigned the following values: TBS-1 and TBS-2 were assigned a value of 0; TBS-3 and TBS-4 were assigned a value of 1; TBS-5 and TBS-6 were assigned a value of 2.

### 2.4 BRAF V600E mutation analysis

The gene mutation detection and analysis process based on real-time fluorescent quantitative PCR technology is as follows. The FNA sample was thoroughly mixed with the premixed reaction solution (a 45 *µ*L reaction system) and 0.6 *µ*L of Taq enzyme, and then dispensed into centrifuge tubes containing the DNA template. Thermal cycling was performed using the ABI Quant Studio Dx fully automated nucleic acid amplification system (Applied Biosystems, USA), with reaction parameters strictly set according to the manufacturer’s recommendations. The criteria for result interpretation were as follows: if the fluorescein amidite (FAM) channel of the detection well exhibited a typical amplification curve and the cycle threshold (Ct) value below 30, the sample was confirmed to have the existence of the BRAF V600E mutation; conversely, if no characteristic amplification peak was observed or if the FAM-Ct value was 30 or greater, the sample was considered negative for the BRAF V600E mutation. The entire testing process and interpretation of results were carried out by a single experienced operator, with a quality control standard and a no-template control included in each run.

### 2.5 Gold standard specification

All surgical patients were evaluated using histopathology as the gold standard, and histopathological reports were prepared in accordance with the World Health Organization guidelines(2017 Edition) (22). For these nodules without postoperative histopathology, when the nodule is considered benign in the initial FNAC, it is subjected to a rFNAC six months later after a comprehensive evaluation. If the nodule continues to show benign results during the follow-up, it is classified as benign. Essentially, two consistent benign outcomes from FNAC-based examinations conducted at a six-month interval were considered the gold standard for identifying patients with benign lesions that did not require surgical treatment. Conversely, if the rFNAC result demonstrates malignancy, the patient will undergo further surgery, with subsequent pathological results serving as the gold standard for definitive diagnosis. Each nodule was labeled as either benign or malignant according to the diagnostic gold standard. Within the retrospective data, we trained different machine learning models and internally validated their discrimination and calibration. Additionally, we used prospective data to assess the performance of these models in greater depth. This study included retrospective data and two prospective cohorts. In the retrospective data (n = 620), there are 111 benign nodules and 509 malignant nodules. The benign nodules were confirmed by postoperative pathology in 90 cases and by the second-round FNAC in 21 cases. The malignant nodules consisted of 504 papillary carcinomas, 3 medullary carcinomas, 1 follicular carcinoma, and 1 undifferentiated carcinoma. In the first prospective cohort (n = 243), there are 47 benign nodules and 196 malignant nodules, with benign cases confirmed by postoperative pathology (n = 35) and second-round FNAC (n = 12). The malignancies comprised 194 papillary carcinomas and 2 follicular carcinomas. The second prospective cohort (n = 70, all with BRAF V600E mutation analysis) included 10 benign nodules (5 confirmed surgically, 5 by rFNAC) and 60 papillary carcinomas.

### 2.6 Classification models

In addition to the key factors of FNAC diagnosis and AI-assisted ultrasound evaluation, we incorporated age, sex, maximum diameter, and location of nodules as the complete set of features for model development. The location of thyroid nodules is an independent risk factor for predicting the likelihood of thyroid cancer, with isthmic nodules carrying the highest risk of malignancy (23). The retrospective data was randomly split into a training cohort and a testing cohort (Testing1) in a 7:3 ratio. Using the Training cohort, we trained five different models using five distinct algorithms. To evaluate their performance, we first tested these models using the Testing1 cohort. Furthermore, to assess the stability of these models, we used the first prospective cohort as the Testing2 cohort. Finally, the optimal model was validated using the second prospective cohort (Testing3), in which it was compared with FNAC combined with BRAF V600E mutation analysis. These classification models operate by mapping the features of a single instance and categorizing the data into groups based on attribute values. When presented with a new instance, the model assigns it to the category that optimally matches its learned attributes. The architecture of classification models spans a spectrum, from linear discriminant functions to clustering techniques and ensemble methods, each offering distinct benefits tailored to the specific properties of the dataset it aims to process. Finally, we selected five widely adopted models in the medical diagnostic field to test their discrimination and calibration. The models chosen are introduced as follows:

1. Logistic Regression (LR) (24): LR is used for binary classification problems by mapping the output of a linear combination through a logistic function (sigmoid function) to a value between 0 and 1, thereby predicting the probability of an event.
2. Support Vector Machine (SVM) (25): SVM separates data points of different categories by identifying the hyperplane that maximizes the separation between them. This optimal hyperplane is chosen to ensure the largest margin between the classes. For nonlinear problems, kernel functions can be used to project the data onto a higher-dimensional manifold.
3. K-Nearest Neighbour (KNN) (26): KNN is an algorithm that helps classify new samples by assigning them a score based on a set of calculations done on the entire training dataset. When a new sample is introduced, the algorithm compares its score with the scores of all samples in the training group. It subsequently identifies the training sample that is closest in score, termed the ‘nearest neighbor’. By looking at the category of this neighbor, KNN can classify the new sample accordingly.
4. Random Forests (RF) (24): RF is a powerful ensemble learning algorithm that enhances model performance and robustness by constructing a collection of decision trees and synthesizing their outputs through ensemble methods such as majority voting or averaging.
5. Gradient Boosting Machine (GBM) (27): GBM is a highly effective ensemble algorithm famous for its excellent predictive accuracy, especially when dealing with complex patterns. Similarly, it also constructs a strong model by aggregating multiple weak learners (usually decision trees). GBM constructs decision trees in a sequential manner, with each subsequent tree aiming to correct the residual errors of the preceding trees. This iterative approach allows GBM to capture intricate relationships in the data, making it a popular choice for a variety of prediction tasks.

### 2.7 Statistical analysis and AI model

Statistical analysis and the AI model were conducted using Python 3.12.4. In addition to AI ultrasound diagnosis and FNAC, other clinical parameters included in the AI model were age, sex, whether the nodule is located in the isthmus of the thyroid, and the maximum diameter of the nodule. In the case of a thyroid nodule located at the isthmus, it is coded as 1; otherwise, it is coded as 0. These parameters, along with the final gold standard, were included as features in our model’s dataset. For this analysis, the retrospective data was partitioned into 70% for training, 30% for testing. To optimize these models, we used a grid search algorithm. This method explores multiple dimensions by adjusting each parameter individually to find the best results. To train and validate the model while minimizing biases, we employed k-fold cross-validation. In this process, the training set is divided into 10 equal parts (folds). Each fold is utilized as the validation set in turn, with the remaining 9 folds used for training. This procedure is repeated across 10 iterations, ensuring that each fold is employed as the validation set once. To enhance the reliability of performance estimates, we conducted the k-fold cross-validation process ten times (k = 10). These AI predictive models evaluate the nature of thyroid nodules and quantify it as a probability percentage. If the estimated probability is 50% or higher, the AI model classifies the nodule as positive, which means the thyroid nodule is considered malignant by the AI. It is deemed a true positive if the final histopathological results confirm malignancy. Normally distributed continuous variables were presented as mean ± standard deviation, whereas skewed data were reported as M(IQR). Group comparisons were conducted using the independent samples t-test or the Wilcoxon rank-sum test, depending on data distribution and suitability. The categorical variables were presented as proportions, with statistical analysis conducted using Pearson’s Chi-square test or Fisher’s exact test, subject to the sample size and the distribution of expected frequencies within the cells. Model discrimination was evaluated using a confusion matrix alongside several metrics, comprising the area under the receiver operating characteristic curve (AUC), accuracy, sensitivity, specificity, and F1 Score. For subgroup analyses, where small sample sizes precluded the generation of a meaningful confusion matrix, we calculated the percentage of correctly classified cases. In addition, we evaluated model calibration using the Brier score (28). Since the model developed in this study focuses more on discriminative ability, the calibration curve was not plot. Plot the receiver operating characteristic (ROC) curves and decision curve analysis (DCA) curves for different diagnostic methods, and compare their AUC, accuracy, sensitivity, specificity, and Cohen’s Kappa coefficient. *P <* 0.05 was regarded as statistically significant.

## 3 Results

### 3.1 Baseline characteristics across different datasets

The baseline characteristics for the study cohort are summarized in **Table 1**.

**Table 1:** Baseline characteristics across different datasets.

Nodule features	Training cohort	Testing 1 cohort	*P* value	Testing 2 cohort	*P* value	Testing 3 cohort	*P* value
nodule count	434	186		243		70	
Age (mean ± std)	45.47 ± 11.89	46.52 ± 11.30	0.298	45.86 ± 11.49	0.681	44.37 ± 11.96	0.476
Sex (n, %)			0.087		0.003		0.896
Female	323 (74.42)	151 (81.18)		206 (84.77)		51 (72.86)	
Male	111 (25.58)	35 (18.82)		37 (15.23)		19 (27.14)	
Max diameter (mm) M(IQR)	8.00 (7.00)	8.00 (5.00)	0.500	8.00 (5.60)	0.065	7.00 (5.97)	0.053
Location (n, %)			0.968		0.796		0.268
right or left lobes	411 (94.70)	177 (95.16)		232 (95.47)		69 (98.57)	
isthmus	23 (5.30)	9 (4.84)		11 (4.53)		1 (1.43)	
AI ultrasound diagnosis (%) M(IQR)	91.00 (29.00)	90.50 (26.75)	0.283	89.00 (32.50)	0.921	89.50 (19.00)	0.738
Bethesda classification (n, %)			0.514		0.306		0.128
I/II	49 (11.29)	19 (10.22)		25 (10.29)		8 (11.43)	
III/IV	52 (11.98)	30 (16.13)		42 (17.28)		15 (21.43)	
V/VI	333 (76.73)	137 (73.66)		176 (72.43)		47 (67.14)	
Gold standard (n, %)			0.615		0.572		0.653
Benign	75 (17.28)	36 (19.35)		47 (19.34)		10 (14.29)	
Malignancy	359 (82.72)	150 (80.65)		196 (80.66)		60 (85.71)	

### 3.2 Performance analysis of AI models

**Figure 3:**
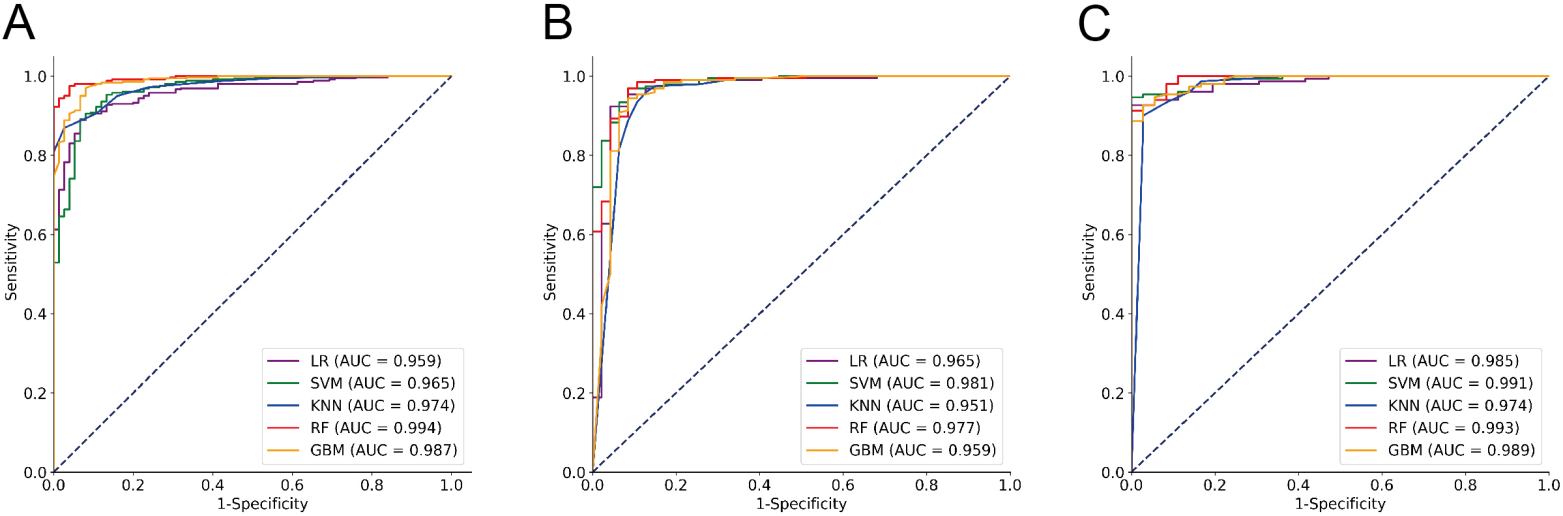
ROC analysis of the performance of five classifier algorithms. **(A)** Training cohort; **(B)** Testing1 cohort; **(C)** Testing2 cohort.

Among these models, the RF model demonstrated the best discrimination in the Training cohort, with an accuracy of 96.54%, sensitivity of 99.16%, specificity of 84.00%, F1 Score of 97.94%, and an AUC of 0.994. It also maintained excellent performance in the Testing1 cohort, achieving an accuracy of 97.31%, sensitivity of 99.33%, specificity of 88.89%, F1 Score of 98.35%, and an AUC of 0.993. Furthermore, we conducted a prospective analysis, and the results show that the RF model is still the best model. From the perspective of calibration, the RF model had the lowest Brier score (Training cohort: 0.030; Testing1 cohort: 0.035) among all models, in other words, the RF model had the best calibration among all models. In terms of longitudinal results, the RF model demonstrated good learning performance on the Training cohort, with no signs of overfitting, allowing it to maintain excellent performance in the prospective study. The GBM model, another ensemble learning model, showed balanced and favorable discrimination in both the Training and Testing1 cohorts, though slightly inferior to the RF model. In contrast, the KNN model and the SVM model demonstrated moderate discrimination. The LR model’s sensitivity, specificity, and F1 Score were lower than those of the other models. It is worth noting that the SVM model has the highest AUC (0.981) and the lowest Brier score (0.040) in the Testing2 cohort, but its accuracy, sensitivity, specificity, and F1 Score are clearly inferior to those of the RF model. Details can be found in **Table 2** and **Figures 3A–C**.

**Table 2:** Performance analysis of AI models in different cohorts.

Model	Accuracy (%)	Sensitivity (%)	Specificity (%)	F-Score (%)	AUC	Brier Score
Training Cohort						
LR	91.24	95.82	69.33	94.77	0.959	0.061
SVM	93.09	97.49	72.00	95.89	0.965	0.050
KNN	93.50	97.21	76.00	96.14	0.974	0.050
RF	96.54	99.16	84.00	97.94	0.994	0.030
GBM	95.62	98.61	81.33	97.39	0.987	0.037
Testing1 Cohort						
LR	93.01	98.00	72.22	95.77	0.985	0.046
SVM	94.62	98.67	77.78	96.73	0.991	0.038
KNN	95.70	98.67	83.33	97.37	0.974	0.039
RF	97.31	99.33	88.89	98.35	0.993	0.035
GBM	95.16	97.33	86.11	97.01	0.989	0.040
Testing2 Cohort						
LR	95.47	98.98	80.85	97.24	0.965	0.042
SVM	94.65	97.96	80.85	96.73	0.981	0.040
KNN	95.06	97.45	85.11	96.95	0.951	0.046
RF	96.71	98.47	89.36	97.97	0.977	0.041
GBM	94.24	96.43	85.11	96.43	0.959	0.045

### 3.3 Performance analysis of the RF model

**Figure 4:**
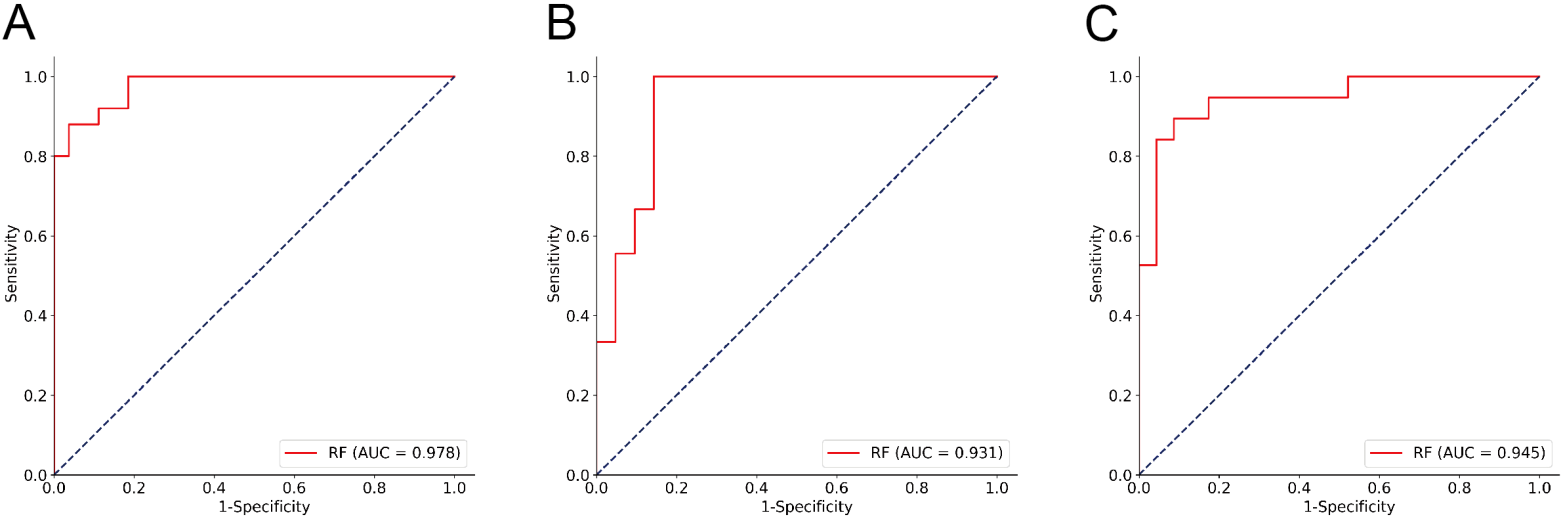
ROC analysis of the RF Model performance for ITNs. **(A)** Training cohort; **(B)** Testing1 cohort; **(C)** Testing2 cohort.

**Figure 5:**
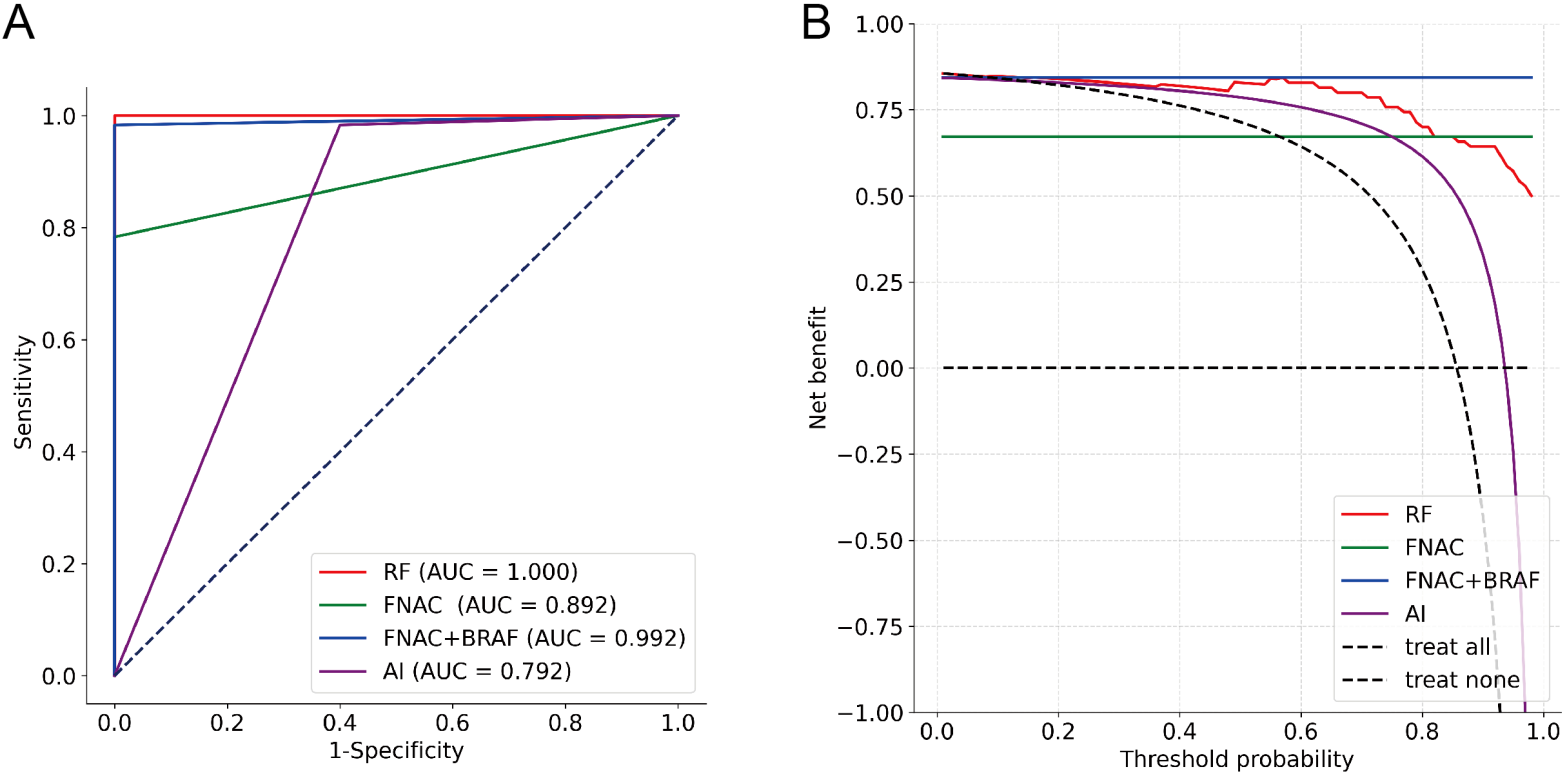
Diagnostic performance evaluation of different methods (n=70). **(A)** ROC curves with AUC values; **(B)** DCA showing net benefit across threshold probabilities.

To further explore the performance of the RF model, we assessed its diagnostic capability for ITNs. When applied to these diagnostically challenging nodules, the developed RF model achieved an accuracy of 90.48%. This satisfactory result suggests that the RF model has robust discriminative capability for ITNs. Detailed information is presented in **Table 3** and **Figures 4A–C**. To validate the diagnostic efficacy and clinical practicality of the RF model, we conducted a comparative analysis with FNAC combined with BRAF V600E mutation analysis. The RF model demonstrated specificity of 100.00%, sensitivity of 98.33%, and a Kappa value of 0.944. Although the specificity of the RF model was 20% lower than that of the combined method, this difference was not statistically significant (*P* = 0.480). Similarly, the Kappa value was 0.071 lower than the combined method, with no statistical significance (*P* = 0.141). Complete data are shown in **Table 4** and **Figure 5A**. Notably, the RF model exhibited superior diagnostic performance compared to the AI ultrasound auxiliary diagnostic system. Meanwhile, the diagnostic efficacy of FNAC alone was significantly compromised by the presence of ITNs. **Figure 5B** displays the DCA curves of different diagnostic methods. Since both FNAC and FNAC combined with BRAF V600E mutation analysis are binary categorical data (0 and 1), their curves appear as horizontal lines parallel to the x-axis. This precluded direct comparison between the RF model and FNAC combined with BRAF V600E mutation analysis. Nevertheless, the RF model demonstrated significantly better performance compared to both the standalone AI ultrasound auxiliary diagnostic system and FNAC alone.

**Table 3:** Performance of the RF Model for ITNs.

Performance metric	Training cohort	Testing1 cohort	Testing2 cohort
Accuracy (%)	90.38	86.67	90.48
Sensitivity (%)	92.00	100.00	89.47
Specificity (%)	88.89	80.95	91.30
F1 Score	0.902	0.818	0.895
AUC	0.978	0.931	0.950

**Table 4:** Comparison of diagnostic performance across different methods (n = 70).

Methods	Malignancy (n, %)	Benign (n, %)	Sensitivity (%)	Specificity (%)	Accuracy (%)	AUC	Kappa value
Gold Standard	60 (85.71)	10 (14.29)	–	–	–	–	–
RF	62 (88.57)	8 (11.43)	100.00	80.00	97.14	1.000	0.873
AI	63 (90.00)	7 (10.00)	98.33	60.00	92.86	0.792	0.667*
FNAC	47 (67.14)	23 (32.86)	78.33*	100.00	81.43*	0.892	0.508*
FNAC+BRAF	59 (84.29)	11 (15.71)	98.33	100.00	98.57	0.992	0.944

*P value *<* 0.05, The RF model was individually compared with AI, FNAC, and FNAC+BRAF.

### 3.4 Interpretability analysis of the RF model

**Figure 6:**
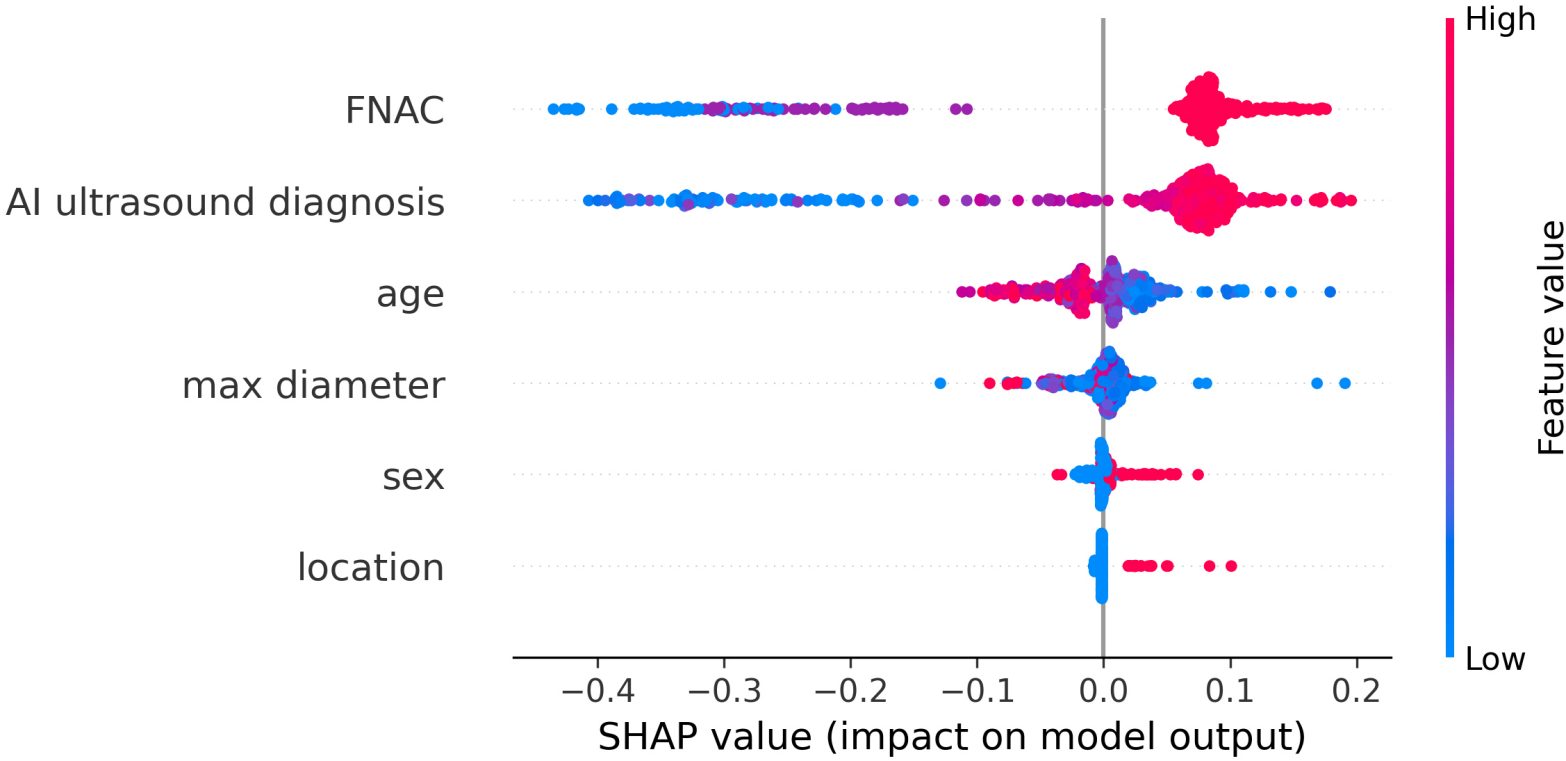
SHAP summary plot of the RF model.

To better understand the mechanism by which the RF model differentiates benign thyroid nodules from malignant ones, the RF model is visually interpreted using Shapley Additive Explanation (SHAP) methods. **Figure 6** presented a comprehensive and detailed swarm plot, demonstrating the key variables in the RF model and providing a clear visualization of their relationships. The horizontal axis represents the SHAP values, while the vertical axis sorts the features according to their cumulative impact, illustrating how each feature contributes to the model’s predictions. Each dot reflects a specific instance, where the feature and instance determine the dot’s position along the x-axis. FNAC and AI ultrasound diagnosis are the two most important factors in diagnosis.

In terms of feature importance, FNAC and AI ultrasound diagnosis ranked highest, followed by age, max diameter, sex, and location. FNAC and AI ultrasound diagnosis are the two most important factors in diagnosis, both contributing positively to the diagnostic outcome. Younger and smaller nodule size also showed positive SHAP values, indicating an association with higher malignancy risk. Conversely, older and larger nodule size were associated with lower predicted probabilities of malignancy. This may be explained by the clinical observation that elderly patients with benign thyroid nodules often undergo surgery due to compressive symptoms from larger lesions. Furthermore, male and isthmus-located nodules exhibited positive contributions to the diagnosis, while SHAP values for female and nodules located in the bilateral lobes were close to zero, suggesting limited impact. These results align well with clinical practice and previously reported findings.

## 4 Discussion

Accurate evaluation of the benign or malignant nature of thyroid nodules is crucial for the clinical diagnosis and therapeutic intervention of thyroid cancer. While most thyroid nodules can be clearly diagnosed through ultrasound and FNAC, there remains a minority that cannot be definitively diagnosed and often requires further diagnostic procedure, such as genetic testing or diagnostic hemithyroidectomies. However, this incurs substantial time and financial costs, as well as the potential need for additional FNAC, which may lead to increased discomfort for the patients. A reliable and non-invasive tool to predict nodule malignancy would significantly assist non-thyroid specialist in evaluating nodules for appropriate referrals, while also aiding thyroid surgery specialist in preoperative assessment of nodule malignancy to minimize unnecessary medical intervention.

In the present research, we developed five AI models to evaluate the malignancy risk of thyroid nodules using the AI-assisted ultrasound diagnostic system, FNAC, and demographic data, and selected the best performing model. Overall, the RF model demonstrated superior performance, prompting further analysis and interpretation. Random Forest is an advanced ensemble learning algorithm that aggregates the outputs of numerous individual decision trees, reducing bias and variance. It randomly selects a subset of features at each node split, decreasing model correlation and minimizing the risk of overfitting. This method effectively handles high-dimensional data and identifies feature importance, optimizing performance. Additionally, Random Forest is robust to outliers and noise, maintaining good performance under complex data distributions. Its ensemble nature also provides built-in cross-validation during training, enhancing generalization.

The RF model that we developed accurately predicted 181 out of 186 nodules in the Testing1 cohort. Among the 5 errors, 1 was false negative predictions. The thyroid incidentaloma was classified as ACR TI-RADS 5, and the size of the nodule was 4.3 × 4.5 × 7.9 mm. The ultrasound also indicated lymphadenopathy in the VI level of the neck, with the enlarged lymph nodes showing no hilar echogenicity, along with features of Hashimoto’s thyroiditis. FNAC was performed, which illustrated lymphocytic thyroiditis. Three months later, a follow-up ultrasound demonstrated an increase in the size of the nodule, prompting a repeat FNAC, which revealed papillary thyroid carcinoma associated with lymphocytic thyroiditis. The patient underwent surgical intervention, and the histopathology confirmed papillary thyroid carcinoma with chronic lymphocytic thyroiditis. The first FNAC diagnosis reviewed again demonstrates that scattered follicular epithelial cells are present in the background of inflammatory cells. The reason for the misdiagnosis may be that the lesion was not accurately sampled. Of the four false positive predictions, three TBS-4 nodules were misclassified as malignant, with histopathology showing non-invasive follicular thyroid neoplasm with papillary-like nuclear features (NIFTP), adenoma, nodular goiter, and one atypical hyperplastic lesion of the thyroid nodule. In the Testing2 cohort (n = 243), the RF model accurately predicted 235 out of 243 nodules. The 8 misclassified nodules included 5 false positives and 3 false negatives. Among the five false positive cases, three nodules initially diagnosed as suspicious for malignancy by FNAC were ultimately confirmed through histopathological examination to be atypical hyperplastic lesions of the thyroid. Notably, FNAC diagnoses categorized as suspicious for malignancy or definitive malignancy significantly influence the predictive outcomes of the RF model. The remaining two false positive cases, which had FNAC diagnoses suggestive of oncocytic neoplasms, were similarly identified as atypical hyperplastic lesions upon histopathological evaluation. There were three false negative cases with histopathological confirmation: one demonstrating follicular carcinoma, one papillary carcinoma confirmed by immunohistochemical assessment, and one papillary thyroid carcinoma associated with Hashimoto’s thyroiditis.

Due to the small sample size of the ITNs subgroup, there are indeed fluctuations in model performance metrics observed in both the Testing1 and Testing2 cohorts with ITNs. However, the RF model demonstrated satisfactory clinical utility across both datasets, showing effective potential to avoid diagnostic surgeries. In the Testing1 cohort with ITNs (n = 30), the RF model accurately identified all malignant nodules (n = 9), while there were a total of 21 benign nodules. Had the RF model been applied to this cohort, 17 out of 21 diagnostic hemithyroidectomies (80.95%) performed on cases diagnosed as benign according to surgical histology could have been averted. Excluding ITNs, the classifiers correctly predicted almost 100% of the cases. In the Testing2 cohort with ITNs (n = 42), there were 11 malignant nodules and 23 benign nodules. Among the 23 benign nodules, the RF model correctly identified 21, preventing further operation. Among the Testing 1 and Testing 2 cohorts with ITNs, analysis of 27 TBS-3 nodules revealed 3 false-positive cases, achieving a correct classification rate of 88.89% (24/27). In comparison, evaluation of 45 TBS-4 nodules identified 5 false-positive instances, demonstrating a diagnostic accuracy of 91.11% (41/45) for correct classification. In the Training, Testing 1, and Test 2 cohorts of this study, TBS-5 nodules exhibited a remarkably high malignancy rate of 95.28% (303/318), which explains why ITNs in this study excluded TBS-5 nodules.

The RF model demonstrates excellent predictive capability, effectively guiding non-thyroid specialists in the preliminary assessment of nodules and assisting thyroid surgeons in predicting the malignancy of ITNs. Among the ITNs, limited cost-effectiveness evidence and accessibility challenges have constrained mutation analysis applications in resource-limited settings, where 70% are benign upon final pathology, potentially leading to unnecessary surgeries (29). The developed classifier provides effective and straightforward differentiation between benign and malignant nodules, which carries significant clinical implications. Unfortunately, this study has several limitations. First, the predominantly postoperative composition of the study cohort introduces selection bias, as this population fails to adequately represent the broader spectrum of thyroid nodule patients, consequently skewing the benignto-malignant ratio. Regarding this issue, a recent study introduced the 2e diagnostic criteria for diagnosing thyroid nodules (30). This criterion follows a two-level hierarchy: in addition to the conventional pathological result (FNAC or postoperative pathological examinations), another level of hierarchy is the decision of an arbitration group consisting of 3 senior ultrasound specialists. Adopting the 2e diagnostic criteria enables the inclusion of a substantial number of benign thyroid nodules in the study cohort, rather than limiting participation to patients with confirmed pathologies, thereby effectively minimizing selection bias. Second, the AI-assisted ultrasound diagnosis is based on retrospective image data. The ITS100 can also perform dynamic AI diagnostics, demonstrating similar diagnostic efficacy (15). Future studies should focus more on dynamic AI diagnostics, making the AI diagnostic system more accessible in regions with limited medical resources for thyroid nodule diagnosis.

## 5 Conclusion

In the present study, we have developed a pilot AI model capable of precisely predicting malignancy of thyroid nodules by integrating the AI-assisted ultrasound diagnosis system, cytology, and demographic information. With further development and refinement for clinical applications, this AI model holds significant promise as a computer-aided decision support tool for non-thyroid specialists and can also assist thyroid surgeons in determining the next steps in the management of ITNs.

## Data Availability

The original contributions presented in the study are included in the article/Supplementary Material. Further inquiries can be directed to the corresponding author.
